# Defensive eye-blink startle responses in a human experimental model of anxiety

**DOI:** 10.1177/0269881114532858

**Published:** 2014-06-04

**Authors:** Verity Pinkney, Robin Wickens, Susan Bamford, David S Baldwin, Matthew Garner

**Affiliations:** 1 Psychology, University of Southampton, Southampton, UK; 2 Department of Pharmacy and Pharmacology, University of Bath, Bath, UK; 3 Clinical and Experimental Sciences, University of Southampton, Southampton, UK

**Keywords:** Anxiety, carbon dioxide inhalation, defensive behaviour, emotional response, eye-blink startle response, heart rate, skin conductance, startle

## Abstract

Inhalation of low concentrations of carbon dioxide (CO_2_) triggers anxious behaviours in rodents via chemosensors in the amygdala, and increases anxiety, autonomic arousal and hypervigilance in healthy humans. However, it is not known whether CO_2_ inhalation modulates defensive behaviours coordinated by this network in humans. We examined the effect of 7.5% CO_2_ challenge on the defensive eye-blink startle response. A total of 27 healthy volunteers completed an affective startle task during inhalation of 7.5% CO_2_ and air. The magnitude and latency of startle eye-blinks were recorded whilst participants viewed aversive and neutral pictures. We found that 7.5% CO_2_ increased state anxiety and raised concurrent measures of skin conductance and heart rate (HR). CO_2_ challenge did not increase startle magnitude, but slowed the onset of startle eye-blinks. The effect of CO_2_ challenge on HR covaried with its effects on both subjective anxiety and startle latency. Our findings are discussed with reference to startle profiles during conditions of interoceptive threat, increased cognitive load and in populations characterised by anxiety, compared with acute fear and panic.

## Introduction

Inhalation of air enriched with 7.5% carbon dioxide (CO_2_) produces reliable increases in subjective anxiety and autonomic arousal (e.g. blood pressure and heart rate (HR)) in healthy humans (Bailey et al., 2005). The subjective effects of 7.5% CO_2_ challenge are well characterised, and include increased anxiety, nervousness, worry, fearful apprehension and tension (Bailey et al., 2005). These feelings are quantitatively and qualitatively less pronounced than the sudden acute feelings of panic (overwhelming intense fear and discomfort) that accompany the single vital capacity inhalation of 35% CO_2_ (Colasanti et al., 2008). Accordingly, there is growing consensus that 7.5% CO_2_ challenge provides an experimental model of anxiety that complements, but differs from the 35% CO_2_ model of panic.

Recent research has examined whether 7.5% CO_2_ challenge can induce biases in cognition and emotion processing that promote the feelings of worry, nervous apprehension and perceptions of threat that characterise anxiety. For example, 7.5% CO_2_ challenge increases attention (erroneous eye-movements) to threatening aversive, visual stimuli in an antisaccade task (Garner et al., 2011) and increases hypervigilance through enhancing alerting (temporal) and orienting (spatial) attention network function (Garner et al., 2012). Similarly, studies in rodents show that exposure to 10% CO_2_ increases behavioural inhibition, freezing and reduced activity in an open-field test (Ziemann et al., 2009). Thus, across species, inhalation of low concentrations of CO_2_ appears to trigger a range of behavioural responses characteristic of the anxiety phenotype.

Adaptive responses to threat can be considered across a defence cascade. An anxious preparatory state is characterised by vigilance, alertness, behavioural inhibition and appraisal, and enables the organism to monitor the risk associated with an anticipated, distal, often uncircumscribed threat. In contrast, active defence and avoidance (fight-flight) characterise an acute fear state that is mobilised by an identified, localized and proximal threat (for extended discussion of fear versus anxiety in humans and rodents see Blanchard et al., 2001; McNaughton, 2011; McNaughton and Corr, 2004).

One of the most reliable components of human defence is the eye-blink reflex, a rapid and intense contraction of the orbicularis muscle in response to a startling stimulus (typically a loud noise). This defensive reflex is greater (potentiated) in threatening contexts (e.g. when the delivery of an aversive shock is unpredictable; Grillon et al., 2004; Grillon et al., 2006), and when viewing aversive pictures (Smith et al., 2005; Vrana et al., 1988). Conversely, the startle response is reduced to positive/appetitive stimuli (e.g. Vrana et al., 1988). Startle responses are greater in fearful individuals (e.g. when phobic individuals view fear-provoking stimuli; Hamm et al., 1997) and also in several anxious populations (see Vaidyanathan et al., 2009 for a review) such as with post-traumatic stress disorder (PTSD) (e.g. Morgan et al., 1995) and panic disorder (e.g. Melzig et al., 2007). Comparatively few studies have examined the affective modulation of startle latency, however there is evidence that startle responses are quicker to aversive, relative to positive stimuli (Panayiotou et al., 2011; Witvliet and Vrana, 1995), and to stimuli that elicit high, relative to low levels of arousal (Cook et al., 1991; Hawk et al., 1992; Witvliet et al., 1995).

Research in rodents (Hitchcock and Davis, 1986, 1991; Rosen et al., 1991), human imaging (Pissiota et al., 2003) and human lesion studies (Buchanan et al., 2004; Funayama et al., 2001) implicate the extended amygdala, and in particular the central nucleus of the amygdala and the bed nucleus of the stria terminalis (BNST), in mediating startle potentiation. Lesion studies suggest that the central nucleus of the amygdala potentiates startle responses to brief, short-duration fear-provoking aversive stimuli (i.e. it mediates fear-potentiated startle). Conversely, the BNST does not potentiate startle to discrete aversive cues, but does potentiate startle over sustained periods of anxiety (for example, when nocturnal rodents are exposed to bright light or when humans anticipate prolonged uncertain threat; see Grillon, 2008 for a review). Likewise, the central role of the extended amygdala in normal fear and pathological anxiety is well characterised (Davidson, 2002; Davis and Whalen, 2001). Furthermore, recent evidence in rodents suggests the amygdala functions as an important chemosensor that directly detects increases in CO_2_ (via acid-sensing ion channels (ASIC1a)), to increase behavioural inhibition and freezing in rodents (Ziemann et al., 2009).

While inhalation of low concentrations of CO_2_ increases anxiety and autonomic arousal in humans (Bailey et al., 2005), and triggers anxious behaviour in both small animals (Ziemann et al., 2009) and humans (Garner et al., 2011, 2012), its effects on the defensive behaviours that are mediated by the extended amygdala are not known. To date, only two studies have explored the effects of CO_2_ challenge on the human eye-blink startle response (Ceunen et al., 2013; Pappens et al., 2012). Both studies examined the magnitude (but not latency) of three startle responses to acoustic probes delivered during a short (< 2 min) inhalation of 7.5% CO_2_. Contrary to predictions, startle magnitudes were reduced (rather than potentiated) during CO_2_ challenge, relative to baseline. These findings contrast with evidence that 7.5% CO_2_ challenge over longer durations (10–20 minutes) can *increase* anxious behaviour in response to threat in both humans and animals.

We compared the effects of 7.5% CO_2_ versus air inhalation on eye-blink startle reactivity to threatening (aversive) and non-threatening (neutral) picture stimuli. An optimal adaptive startle response to threat should be both robust and quick. Our study is the first to examine the effect of 7.5% CO_2_ challenge on both startle magnitude and startle latency. We predicted that if 7.5% CO_2_ inhalation triggers defensive behaviour coordinated by the amygdala, then eye-blink startles would be larger and faster during CO_2_, and particularly in response to aversive images.

## Methods

### Ethical considerations

This study was approved by the University of Southampton Ethics and Research Governance Committee in the UK. All participants provided written informed consent prior to participation.

### Research participants

Our study was completed by 27 participants (16 female) aged 18–26 years old (mean age = 20.62, standard deviation (SD) = 2.14). Participants completed a health screen by telephone and a pre-test screening interview in order to confirm their eligibility. Exclusion criteria included: current or history of psychiatric illness as assessed by the MINI International Neuropsychiatric Interview (based on DSM-IV; Sheehan et al., 1998), personal or family history of panic disorder or panic attacks, medication use within the last 8 weeks (apart from local treatment, occasional aspirin or paracetamol, and contraceptives), smoking, history of asthma/respiratory disease, diabetes, migraines, cardiovascular disease, excessive alcohol consumption (this was set at > 50 units/week for males or > 35 units/week for females; the mean intake across eligible participants was 9.4 units/week, SD = 6.9) or a positive alcohol breath test, current or past alcohol or drug dependence (including recent recreational drug use), being under- or over-weight (body mass index (BMI) < 18 or > 28kg/m^2^), blood pressure exceeding 140/90 mmHg or a HR of < 50 bpm or > 90 bpm, caffeine consumption of > 8 caffeinated drinks/day, or pregnancy/breastfeeding. Levels of trait anxiety (trait version of the State–Trait Anxiety Inventory (Spielberger et al., 1983); mean = 32.10, SD = 6.67) were comparable with those observed in healthy control groups (Garner et al., 2009).

### Procedure

Participants attended a single test session and completed an affective startle task twice: once during a 20-minute inhalation of air enriched with 7.5% CO_2_ (a balance of 7.5% CO_2_, 21% O_2_ and 71.5% N_2_) and once during a 20-minute inhalation of normal air. Inhalations were administered blind to participants and were separated by a 30-minute break to remove potential carry-over effects. The gas was administered through an oro-nasal face mask with the inhalation order (i.e. CO_2_ versus air first) counterbalanced across participants in a within-subjects, single blind, cross-over design.

Measures of subjective state anxiety (Spielberger et al., 1983), positive and negative affect (Watson et al., 1988), and blood pressure (Omron-M6 arm-collar, Medisave, UK) were taken at the pre-test baseline (10 minutes before the first inhalation) and immediately (within 1 minute) after each inhalation period. Subjective ratings reflected the ‘peak effects’ of each inhalation. HR was measured at baseline (via the arm-collar) and recorded throughout each 20-minute inhalation from two electrodes placed on both wrists. An electrocardiogram (ECG) was recorded at 1000 Hz with a MP150 amplifier and AcqKnowledge 4.1 software (Biopac, CA, USA).

### Startle task

The startle task took 8 minutes to complete and was administered 2 minutes after each 20-minute inhalation period began. Participants were instructed that they would see a series of pictures and hear occasional noises. Participants viewed 32 images (16 aversive and 16 neutral) taken from the International Affective Picture Set (IAPS) (Lang et al., 2005). The images were selected on the basis of normative valence (on a scale of −4 to +4) and image arousal ratings (0–8; for aversive images the mean valence was −3.85 and the mean arousal was 6.74; for neutral images, the mean valence was 2.30 and the mean arousal was 4.24). Images subtended 22.2 × 15.1 visual degrees (viewed at 58 cm distance) and were presented using Inquisit 2 (Millisecond.com, 2002).

Startle reflexes were elicited with a 50ms, 96dB burst of white noise with a near instantaneous rise/fall time, delivered via headphones. A familiarisation block of 3 habituation startle probes was followed by an experimental block comprising 32 randomly ordered trials (24 experimental picture startle trials, 4 inter-trial interval (ITI) startle trials and 4 no-startle trials). On the experimental trials, aversive and neutral images were presented for 4000ms. The startle probe was presented 3000ms after the image onset. Interspersed within the experimental trials were four no-startle trials where the startle probe was omitted, and four trials where the startle probe was presented 7000ms after picture offset during a 14-second ITI. Both ITI- and no-startle trials were included to reduce the predictability of the startle probe. Picture valence was counterbalanced across trial type.

Eye-blink electromyography (EMG) data were recorded using two 4 mm electrodes placed under the centre and the outer canthus of the right eye. EMG was sampled at 1000 Hz, amplified by 10,000, rectified, filtered (30–500 Hz) and integrated (20 ms constant) using a Biopac MP150 data acquisition system and AcqKnowledge 4.1 software. Skin conductance responses to images were also recorded with silver/silver chloride (Ag-AgCl) electrodes and conductive gel attached to the medial phalanges of the ring and middle fingers of the participant’s non-dominant hand.

Following the startle task, participants completed a 7-minute behavioural measure of impulse-control; see the stop-signal reaction time (SSRT) task. In this task, each participant’s SSRT is estimated from a staircase analysis of their reaction-time distribution. For several participants, the algorithm was unable to converge on a reliable estimate of SSRT for both inhalations, perhaps reflecting an insufficient number of trials in our version of the task. Thus, reliable SSRT data was not available for this report.

### Data preparation

#### Startle magnitude and latency

Data from four participants were excluded from all startle analyses: three due to a technical fault (recording failure) and one who did not complete the task in full. Inspection of boxplots revealed startle responses that occurred < 50 ms as extreme outliers. This equated to 3.3% of the experimental trials and these data were removed from both the magnitude and latency analyses. Startle magnitude was defined as the maximum response between 50–120ms after probe onset minus the mean EMG activity during the 50ms prior to probe onset. To correct for inter-subject variability, all blink magnitudes were standardised to T-scores (i.e. ((*z* × 10) +50)) within each participant using the condition mean and SD, which is a common procedure (Blumenthal et al., 2005). Startle latencies were reported relative to probe onset.

#### Skin conductance

Skin conductance responses (SCRs) to the pictures were calculated by subtracting the mean skin conductance level 1000ms before picture onset (pre-trial baseline SCR) from the maximum skin conductance level between the 900–4000ms window after picture onset (peak SCR); this window excluded SCR responses to the acoustic startle probes.

#### Heart rate

ECGs were band pass filtered (0.5–35 Hz) and QRS-template matched using AcqKnowledge 4.1 software.

## Results

### Effects of 7.5% CO_2_ inhalation on subjective mood and cardiovascular function

Inhalation of 7.5% CO_2_ significantly increased state anxiety and HR, and decreased positive affect (Table 1). Systolic and diastolic blood pressure were elevated during both CO_2_ and air inhalation, relative to baseline (see Table 1).

**Table 1.** Effects of 20-minute 7.5% CO_2_ challenge on anxiety, mood and autonomic arousal.

	Baseline		Air		7.5% CO_2_		ANOVA		
	*M*	*SD*	*M*	*SD*	*M*	*SD*	*F*	*p*	*N_p_^2^*
State anxiety	30.88^a^	(9.27)	33.75^a^	(8.72)	39.83^b^	(10.40)	15.87	.001	.379
Positive affect	30.00^a^	(7.91)	28.46	(7.80)	25.78^b^	(7.45)	7.69	.001	.228
Negative affect	12.26	(3.58)	12.48	(3.66)	13.63	(5.10)	2.36	.133	.079
Systolic BP	117.52^a^	(13.43)	124.60^b^	(17.95)	131.28^b^	(22.30)	14.00	.001	.368
Diastolic BP	70.60^a^	(7.42)	75.52^b^	(7.26)	75.76^b^	(12.12)	7.15	.005	.230
Heart Rate	71.92^a^	(11.09)	72.99^a^	(9.70)	78.43^b^	(12.65)	10.63	.001	.316

Within each variable (row), the values with different superscripts are significantly different from each other: *p* < .017 (Bonferroni correction applied).

ANOVA: analysis of variance; BP: blood pressure; CO2: carbon dioxide; M: mean; SD: standard deviation; N_p_^2^: partial eta squared; F: test statistic for analysis of variance.

### Effect of 7.5% CO_2_ on startle and skin conductance responses

Within each dependent measure, repeated measures analyses of variance (ANOVA) examined the effects of inhalation (7.5% CO_2_ versus air), picture valence (aversive versus neutral) and their interaction, on startle magnitude, startle latency and skin conductance response (see Table 2 for descriptive statistics). There were no significant effects on startle magnitude (*F*’s < .274; *p*’s > .61). Startle latency was significantly slower during the inhalation of 7.5% CO_2_ relative to air, where *F* (1, 22) = 5.38; *p* = .030; η_p_^2^ = .196 (*M* = 102.22; *SE =* 0.95 and *M* = 100.71; *SE =* 0.92, respectively). Skin conductance responses were significantly greater during inhalation of 7.5% CO_2_ (*M* = .085; *SE* = .019) than air (*M* = .024; *SE* = .012; *F* (1, 25) = 8.85; *p* = .006; η_p_^2^ = .261). All other results were non-significant.

**Table 2.** The untransformed mean (SD) of startle magnitude, startle latency and skin conductance responses during inhalation of air and CO_2_.

	Air		7.5% CO_2_	
	M	SD	M	SD
Startle magnitude (μV)	17.40	(17.70)	17.21	(16.16)
Negative	17.55	(17.56)	17.37	(17.05)
Neutral	17.25	(18.14)	17.04	(15.55)
Startle latency (ms)	100.71	(4.42)	102.22	(4.55)
Negative	100.58	(4.53)	101.75	(5.19)
Neutral	100.83	(4.70)	102.68	(4.16)
Skin conductance (μS)	.0242	(0.06)	.0848	(0.10)
Negative	.0343	(0.11)	.0941	(0.13)
Neutral	.0141	(0.06)	.0756	(0.09)

CO_2_: carbon dioxide; M: mean; SD: standard deviation; uV: startle magnitude; ms: milliseconds; uS: microsiemens.

### Associations between subjective and physiological responses to CO_2_ inhalation

Difference scores were calculated to reflect the degree of CO_2_-induced increases in:

- Subjective response;
- Autonomic response (HR and blood pressure);
- Magnitude and latency of startle response; and
- Skin conductance response.

**Figure 1.**
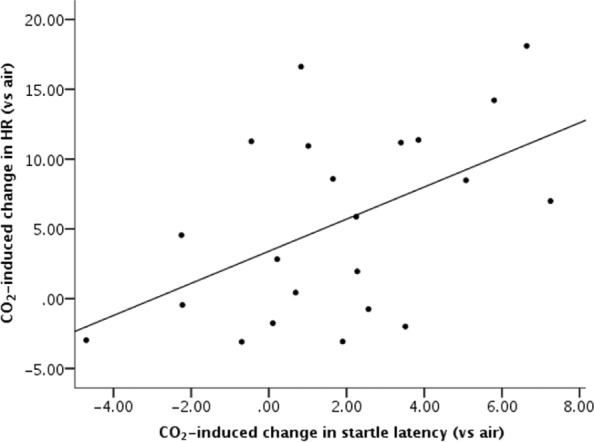
Association between CO_2_-induced increases in heart rate and startle latency. CO_2_: carbon dioxide

There were positive associations between CO_2_-induced state anxiety, HR and blood pressure (Table 3). The effect of CO_2_ on HR was strongly associated with reduced skin conductance responses during CO_2_ inhalation relative to air, and was also associated with slower startle latencies (Figure 1). Furthermore, CO_2_-induced increases in negative affect correlated positively with increased anxiety and HR, whereas CO_2_-induced decreases in positive affect negatively correlated with increased anxiety and blood pressure. Finally, greater negative affect during CO_2_ inhalation was associated with larger startle responses during CO_2_ relative to air.

**Table 3.** Pearson’s R correlations between CO_2_-induced subjective and autonomic responses.

	1.	2.	3.	4.	5.	6.	7.	8.	9.
State anxiety									
Positive affect	−.644^c^								
Negative affect	.771^c^	−.319							
Systolic blood pressure	.497^a^	−.536^b^	.267						
Diastolic blood pressure	.433^a^	−.370	.199	.575^b^					
Heart rate	.461^a^	−.313	.424^a^	.499^a^	.535^b^				
Startle magnitude	.040	.262	.414^a^	−.232	−.241	.179			
Startle latency	.093	−.061	.153	−.082	−.283	.506^a^	.126		
Skin conductance	−.079	−.050	−.116	−.206	−.159	−.452^a^	−.171	.022	

a Significant at < .05

b Significant at < .01

c Significant at < .001

CO_2_: carbon dioxide

## Discussion

The defensive startle response is characterised by a rapid and powerful eye-blink that is potentiated by the extended amygdala. We examined the effects of CO_2_ challenge on both the magnitude and latency of startle responses. Contrary to predictions, 7.5% CO_2_ inhalation did not modulate eye-blink magnitude. Rather, it slowed the latency of eye-blink responses to startle probes. These findings extend previous evidence that inhalation of 7.5% CO_2_ for short periods (<2 minutes) can reduce (rather than potentiate) the magnitude of startle responses to probes that are delivered in the absence of emotional stimuli (Ceunen et al., 2013; Pappens et al., 2012).

Why might 7.5% CO_2_ challenge delay eye-blink startle latencies (present study) and/or reduce their magnitude (Ceunen et al., 2013; Pappens et al., 2012)? One possibility is that CO_2_ challenge may limit the processing resources required for a defensive startle. Consistent with previous findings, CO_2_ challenge produced large increases in subjective anxiety and autonomic arousal, including HR and skin conductance. Furthermore, CO_2_-induced increases in HR covaried with both subjective anxiety and longer startle latency during CO_2_ challenge. Strong positive correlations between CO_2_-increased HR and subjective anxiety were reported in previous studies (Garner et al., 2011, 2012), and likely reflect participants’ use of interoceptive ‘threat’ when rating their subjective anxiety. Notably, attenuated startle responses are observed in paradigms that directly target interoceptive mechanisms (e.g. pain caused by cold pressor or mechanically resisted breathing; Ceunen et al., 2013, Pappens et al., 2011). Startle responses are also attenuated when cognitive load is high (e.g. through increased task demand; see Vytal et al., 2012). Recent comparisons of 7.5% CO_2_ challenge and cognitive load suggest that both manipulations might produce comparable deficits in behaviour through common effects on top-down attention/control mechanisms (Mattys et al., 2013). Thus, CO_2_-induced deficits in cognitive control, together with increased awareness of competing interoceptive threat cues and corresponding increases in cognitive load, may limit the resources required to potentiate startle, thus slowing startle responses and obscuring the typical effects of picture valence on startle magnitude (such as Vrana et al., 1988).

How do our findings fit with those from eye-blink startle studies in other forms of anxiety? Potentiated startle is reliably demonstrated in PTSD (e.g. Morgan et al., 1995), specific phobias (e.g. Hamm et al., 1997), social anxiety (e.g. Cornwell et al., 2006; Garner et al., 2011) and panic disorder (e.g. Grillon et al., 2008). In contrast, there is comparatively weak evidence of potentiated startle in generalised anxiety disorder (GAD; as seen in a review by Vaidyanathan et al, 2009), and even evidence of reduced startle reactivity during anticipation of uncertain threat in GAD relative to other anxiety subtypes (Grillon et al., 2009; see Mcteague and Lang, 2012), which may be due to its high comorbidity with depression where blunted startles are also a common feature (Taylor-Clift et al., 2011).

A 7.5% CO_2_ challenge in healthy volunteers has been proposed as an experimental model of GAD (Bailey et al., 2005, 2011). Drug treatments that are clinically effective for generalised anxiety can reduce some of the deleterious effects of 7.5% CO_2_ challenge and support the GAD model (Diaper et al., 2012). Likewise, we have recently shown that 7.5% CO_2_ challenge can mimic the deficits in attention control that are observed in unchallenged individuals with elevated generalised trait anxiety (Garner et al., 2013). Consequently, the unexpected effects of 7.5% CO_2_ on startle that are reported here appear to be consistent with patterns of startle responding that are observed in conditions associated with broad negative affect, rather than acute periods of fear and panic (McTeague et al., 2012).

Converging evidence implicates the extended amygdala in potentiating startle responses (Pissiota et al., 2003) and mediating CO_2_-induced behaviour in animals (Ziemann et al., 2009). However, our findings and those of Pappens et al. (2012) and Ceunen et al. (2013) suggest that subjective and autonomic response to CO_2_ challenge can occur in the absence of defensive behaviour coordinated by the amygdala. New evidence that individuals with bilateral amygdala lesions can display strong subjective and autonomic responses to 35% CO_2_ challenge suggests that mechanisms beyond the amygdala may mediate the human response to CO_2_ challenge (Feinstein et al., 2013). Esquivel et al. (2010) propose a distributed network of brain regions that underlie CO_2_ challenge, including the locus coeruleus, hypothalamus, midbrain raphe and amygdala. Future research should clarify the neuro-pharmacological networks and peripheral chemoreceptor and mechanoreceptor systems that underlie the subjective, autonomic and behavioural responses to CO_2_ challenge in humans, and the factors that predict individual differences in response to challenge. To this end, research should examine whether anxiolytic drugs that modulate startle during anxious uncertainty but not phasic fear (e.g. the benzodiazepine alprazolam; Grillon, 2008; Grillon et al., 2006) can also reduce the effects of CO_2_ challenge on anxiety, autonomic arousal and startle reactivity. Furthermore, studies should take continuous measures of subjective mood (in addition to peak subjective effects), blood pressure, HR and respiration rate/volume (which was not measured here), to help dissociate phasic and sustained responses throughout CO_2_ challenge. This would extend initial evidence that suggests that the autonomic effects of 7.5% CO_2_ might rise early in the inhalation period and continue to increase gradually across the 20 minutes (Bailey et al., 2005; Poma et al., 2005), and that the effect of CO_2_ on HR in our study was greater during the latter stages of the inhalation (mean HR between 15–20 min of CO_2_ = 81bpm, versus a mean HR between 5–10 min of CO_2_ = 76bpm; *p* = .021; *d* = .88).

Our findings and those of Pappens et al. (2012) and Ceunen et al. (2013) suggest that 7.5% CO_2_ challenge inhibits eye-blink startle. However these three studies differ markedly in design (within versus between subjects), inhalation duration (ranging from < 2 minutes to 20 minutes), number of startles, and affective paradigm (contextual versus emotional picture-potentiated). Our startle paradigm is based on those widely used in previous emotional picture-potentiated startle studies such as by Vrana et al. (1988), however it has not been widely used in within-subject designs, and it is possible that in our study habituation to aversive stimuli may increase the likelihood of Type II error. Future research in this area will benefit from the recent development of standardized startle protocols that have already shown promise in validation studies, and which can differentiate startle responses during phasic cued fear versus sustained contextual anxiety (e.g. the NPU threat test, a standardized protocol consisting of a neutral (N) condition, an aversive condition where the threat is predictable (P) and an aversive condition where the threat is unpredictable (U); see Schmitz and Grillon, 2012). For example, evidence that the effects of 7.5% CO_2_ challenge on startle mimic anxiety-potentiated, rather than fear-potentiated startle in the NPU-threat test would further validate 7.5% CO_2_ as a model of anxiety.

In sum, despite strong effects of 7.5% CO_2_ challenge on subjective anxiety and autonomic arousal, we did not find evidence that CO_2_ challenge potentiates defensive startle behaviour. Instead, findings to date have suggested that 7.5% CO_2_ reduces the speed and magnitude of startle responses, consistent with startle profiles observed during interoceptive threat, increased cognitive load, and in populations characterised by anxiety and depression rather than acute fear and panic.
